# Real-World Data on Thromboprophylaxis in Active Cancer Patients: Where Are We? Are We Getting There?

**DOI:** 10.3390/cancers12071907

**Published:** 2020-07-15

**Authors:** Nikolaos Tsoukalas, Pavlos Papakotoulas, Athina Christopoulou, Alexandros Ardavanis, Georgios Koumakis, Christos Papandreou, Georgios Papatsimpas, Pavlos Papakostas, Georgios Samelis, Charalambos Andreadis, Gerasimos Aravantinos, Nikolaos Ziras, Charalambos Kalofonos, Epameinondas Samantas, Maria Souggleri, Paris Makrantonakis, Georgios Pentheroudakis, Athanasios Athanasiadis, Helen Stergiou, Elli-Sofia Tripodaki, Alexandros Bokas, Anastasios Grivas, Eleni Timotheadou, Evangelos Bournakis, Ioannis Varthalitis, Ioannis Boukovinas

**Affiliations:** 1 401 General Military Hospital, 11525 Athens, Greece; 2 “Theagenio” Anticancer Hospital, 54639 Thessaloniki, Greece; papakotoulas@gmail.com (P.P.); andreadisc@gmail.com (C.A.); lork_nodia@hotmail.com (A.B.); 3 ‘‘Saint Andrew’’ General Hospital, 26335 Patras, Greece; athinachristo@hotmail.com (A.C.); dr.sugleri@gmail.com (M.S.); 4 “Agios Savvas” Anticancer Hospital, 11522 Athens, Greece; ardavanis@yahoo.com (A.A.); gkoumak@otenet.gr (G.K.); ellisophia@hotmail.com (E.-S.T.); tssgrvs@gmail.com (A.G.); 5 “Papageorgiou” General Hospital, 56429 Thessaloniki, Greece; c.papandr@gmail.com (C.P.); timotheadou@auth.gr (E.T.); 6 “IASO” Thessalias Hospital, 41500 Larissa, Greece; g_papatsibas@yahoo.gr; 7 “Metropolitan” General Hospital, 15562 Athens, Greece; p.papakostas@gmail.com; 8 “Ippokrateio” General Hospital, 11527 Athens, Greece; oncologydept.samelis@hippocratio.gr; 9 “AgioiAnargyroi” Anticancer Hospital, 14564 Kifissia, Greece; garavantinos@yahoo.gr (G.A.); epsam@otenet.gr (E.S.); 10 “Metaxa” Anticancer Hospital, 18537 Piraeus, Greece; zirasngr@otenet.gr; 11 General Hospital University of Patras, 26504 Rio, Greece; kalofonos@upatras.gr; 12 “Interbalkan” Medical Center, 55535 Thessaloniki, Greece; pmakrant@gmail.com; 13 General Hospital University of Ioannina, 45500 Ioannina, Greece; gpenther@otenet.gr; 14 General Hospital of Larissa, 41221 Larissa, Greece; athanasiadis.athanasios@gmail.com; 15 “Bioclinic” Hospital, 54622 Thessaloniki, Greece; hesmo@otenet.gr (H.S.); ibouk@otenet.gr (I.B.); 16 “Aretaieio” University Hospital, 11528 Athens, Greece; vagimith@yahoo.com; 17 “Errikos Dunant” Hospital, 11526 Athens, Greece; vari49@gmail.com

**Keywords:** cancer, active cancer, thrombosis, cancer associated thrombosis (CAT), thromboprophylaxis, low molecular weight heparins (LMWHs)

## Abstract

*Background:* Cancer patients are at high risk for cancer-associated thrombosis (CAT). CAT is the second leading cause of death in these patients but it can be preventable with thromboprophylaxis. *Patients and Methods:* An observational, prospective, multicenter study aiming to record CAT management in clinical practice was conducted by the Hellenic Society of Medical Oncology (HeSMO). *Results:* A total of 426 active cancer patients (mean age 65.3 years, mean BMI: 26.1 kg/m^2^) who received thromboprophylaxis, were included from 18 oncology units. Tumor types were lung 25.1%, pancreas 13.9%, breast 8.7%, stomach 8.5%, ovarian 7.8%, and others 36%, while 69% had metastases. A total of 71% had a Khorana score ≤2 and 61% received High Thrombotic Risk Chemotherapy Agents (HTRCAs, e.g., platinum). For thromboprophylaxis patients received mainly Low Molecular Weight Heparins (LMWHs), on higher than prophylactic doses in 50% of cases. Overall, 16 (3.8%) thrombotic events and 6 (1.4%) bleeding events were recorded. Notably, patients on higher doses of LMWHs compared to patients who received standard prophylactic doses had 70% lower odds to develop thrombotic events (OR: 0.3, 95% CI: 0.10–1.0, *p* = 0.04). *Conclusion:* CAT is an important issue in oncology. Along with the Khorana score, factors as metastases and use of HTRCAs should also be taken into consideration. Thromboprophylaxis for active cancer patients with LMWHs, even on higher doses is safe and efficient.

## 1. Introduction

Cancer is strongly associated with thrombosis, as historically reported by Dr. Armand Trousseau [1]. Patients with cancer are at approximately 15% risk for thrombosis [2], while patients with idiopathic VTE have about a 10% risk of being diagnosed with cancer [3]. More than 60% of occult cancers are diagnosed shortly after the diagnosis of unprovoked venous thromboembolism [4,5]. There is a vicious cycle between cancer and factors like tissue factor (TF), with cancer inducing alterations in the hemostatic system and thrombosis, contributing to tumor progression [6]. Cancer cells proliferation and metastasis is driven by the same oncogenes that are responsible for the hemostatic system [7].

Cancer and thrombosis have a direct relationship, with several mechanisms being implicated. All three components of Virchow’s triad, blood flow, endothelial injury, and hypercoagulability, are influenced by cancer [8]. There are numerous factors [9,10,11] with varying risk potential, contributing to the thrombosis burden. In general, these factors can fall into four groups—patient-related factors, tumor-related factors, treatment-related factors [3], and other factors indicated by biomarkers (tissue factor, P-Selectin).

Cancer is an omni-coagulable disease, with recent findings indicating that inflammation plays a key role in tumor progression and survival across several cancer types [12]. Cancer-related inflammation affects many aspects of malignancy, including proliferation, survival, angiogenesis, and tumor metastasis [13]. In addition, inflammation seems to be a part of a triple play along with thrombosis and cancer, and is at the forefront of research in hematology and immunology [14,15,16,17].

Thrombosis constitutes a public health burden, and in 20% of cases is cancer-related. Thrombotic episodes in active cancer patients complicate or delay cancer treatment, worsen prognosis, and add up to their physical and psychological stress, increasing the social and financial burden and impacting the quality of life. Patients with occurrence of VTE during chemotherapy have significantly worse progression-free survival (PFS) and overall survival (OS), compared to patients without such events [18].

However, CAT is preventable with thromboprophylaxis. Among the various risk assessment models, the Khorana risk assessment model is a validated one that associates the type of cancer, hemoglobin level or use of erythropoietin, platelets count, leukocytes count, and body mass index (BMI) with the risk of thrombosis [19]. Khorana risk assessment model calculates a score for the stratification of patients with active cancer to low, intermediate, and high risk for thrombosis. Several guidelines advice for thromboprophylaxis in high-risk active cancer outpatients (Khorana score ≥2 prior systemic chemotherapy), provided that there are no significant risk factors for bleeding and no drug-drug interactions (DDIs) [20].

Real-world data on thromboprophylaxis in active cancer patients are scarce, thrombosis consequences are very important and the guidelines do not yet oblige physicians to follow the proposed prophylactic procedures. For this reason, within the framework of the Greek Management of Thrombosis (GMaT) study, an observational, prospective, multicenter study on management of CAT that was conducted by the Hellenic Society of Medical Oncology (HeSMO), we try to record the antithrombotic prophylaxis clinical practice in active cancer patients.

## 2. Results

### 2.1. Baseline Characteristics of Patients

In total, 426 patients with active cancer who received primary thromboprophylaxis were included in this study. The baseline characteristics of these patients are presented in Table 1, illustrating that there were two large groups of tumor types—lung cancer (25.1%) and gastrointestinal cancers (29.6%).

In terms of other characteristics, 107 (25.2%) were smokers, 115 (27.1%) had comorbidities, 10 patients were treated with tamoxifen, and 41 (11.2%) patients were hospitalized for some period. Additionally, 13 patients (4.32%) had trauma history, 4 (0.9%) had history of heart failure, and 8 (1.9%) had history of thrombosis. History of severe renal insufficiency was reported for 18 patients (4%).

### 2.2. Risk Assissment for CAT

The majority of patients had metastatic disease (69.5%). High Thrombotic Risk Chemotherapy Agents (HTRCAs), such as platinum-based compounds [21], some anti-metabolites [22], and anti-angiogenic factors [21] (Table 2), were administered to 286 (67.1%) patients. As mentioned before, a significant percentage of patients (27.1%) had comorbidities (cardiological, respiratory, and endocrinological issues).

In Table 3, various well-known risk factors for CAT are illustrated, according to cancer, treatment, patient, and biomarker-related risk factors grouping for each primary cancer type. Other non-listed solid tumors included—head and neck (7), melanoma (5), brain (5), liver (6), unknown primary site (11), larynx (5), CNS (4), pelvis (3), sarcomas (8), cervical (4), endometrium (6), kidney (4), cholangiocarcinomas (2), peritoneal mass (2), penis (2), and esophageal (5).

### 2.3. Stratification of Patients According to the Khorana Score

**Figure 1:**
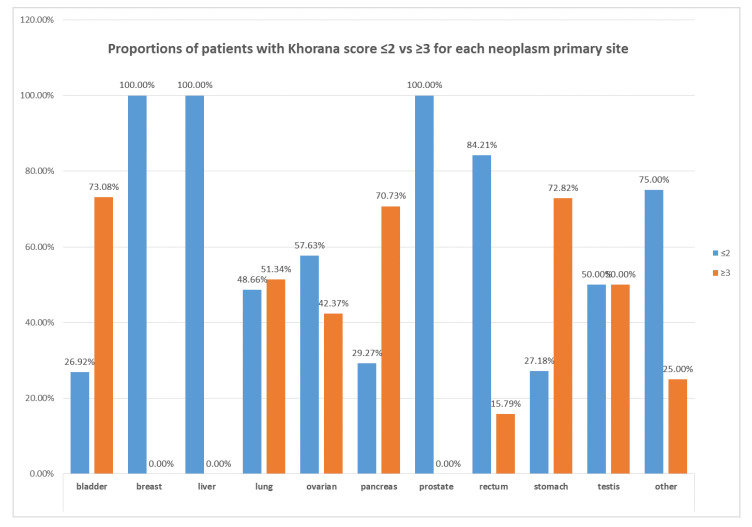
Proportions of patients with Khorana score ≤2 vs. ≥3 in relation to cancer type.

The distribution of cases with Khorana score ≤2 and ≥3, along with the cancer type, is illustrated in Figure 1. About 70% of patients with pancreatic, stomach, or bladder cancer had a Khorana score ≥3. However, the percentage of patients with score ≥3 was not negligible for the other cancer types.

### 2.4. Characteristics of Patients with Khorana Score ≤2

A total of 71% of patients had a Khorana score ≤2, while lung cancer vs. gastrointestinal cancers was about similar (23.7% vs. 22.4%, respectively). Notably, up to 70% had metastatic disease and 61% of the patients received HTRCAs.

For patients with a Khorana score ≤2, 62 (20.4%) were smokers, 76 (25.1%) had comorbidities, 8 patients were treated with tamoxifen, and 31 (12.5%) patients were hospitalized for some period. Additionally, 14 patients (3.3%) had trauma history, 1 (0.3%) had history of heart failure, and 7 (2.3%) had history of thrombosis. The baseline characteristics of this cohort, the cancer types, and the various risk factors appear in the Appendix (Table A1 and Table A2).

### 2.5. Clinical Features Influencing the Decision to Administer Thromboprophylaxis for Patients with Khorana Score ≤2

**Figure 2:**
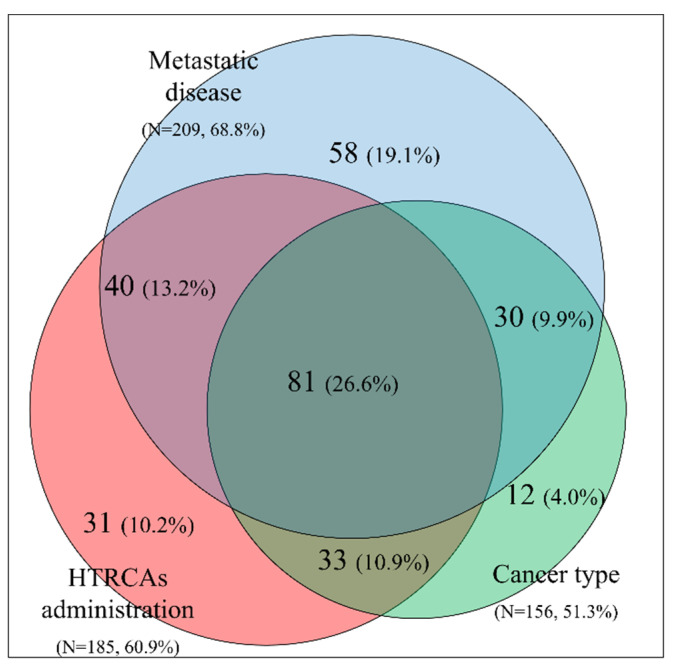
Venn diagram for patients with Khorana score ≤2. The following risk factors are presented: cancer type included in the Khorana risk assessment model (pancreatic, stomach, lung, gynecologic, bladder, testicular), metastatic disease, and treatments with High Thrombotic Risk Chemotherapy Agents (HTRCAs).

In patients with Khorana score ≤2, three important factors that affect the decision to administer thromboprophylaxis were identified: (a) tumor type included in the Khorana score, (b) metastatic disease, and (c) administration of HTRCAs. A graphical representation of the percentages and overlap of these three factors is depicted in the form of a Venn diagram in Figure 2. Within the study cohort, 60.5% of patients with a Khorana score ≤2 had two or three co-existing factors, while 26.6% had three factors.

In an effort to organize the additional risk factors attributing to a higher burden of thrombosis, a four-dimension CAT risk table (Table 4) is presented, illustrating the cancer, treatment, patient, and biomarker-related characteristics, following a similar categorization already published in the literature [23].

### 2.6. Thromboprophylaxis Regiments, Doses and Duration

Majority of active cancer patients received LMWHs (tinzaparin, bemiparin, enoxaparin) and fondaparinux for primary thromboprophylaxis. Tinzaparin was used in 95.3% of the patients and other agents used were—bemiparin (2.0%), enoxaparin (0.5%), and fondaparinux (2.2%). Notably, 50.1% of active cancer patients received higher than prophylactic doses. The mean duration of prophylaxis was 5.0 ± 3.1 months.

No differences in the administered doses were observed between the groups of patients with Khorana score ≤2 and ≥3 (*p* > 0.05). Higher than prophylactic doses were administered in approximately 50% of patients with Khorana scores ≤2. Similarly, 50.8% of patients with Khorana score ≥3 received higher doses. With regards to anticoagulation duration, patients with Khorana score ≥3 received prophylaxis for longer time periods (7.6 ± 5.2 months), compared to patients with Khorana score ≤2, who received anticoagulation for 4.5 ± 3.4 months (*p* < 0.0001).

The univariate statistical analysis with end-point the administration of higher than prophylactic doses, revealed several factors related to the anticoagulation–thromboprophylaxis dose, which seemed to drive or to affect the clinical decision for the administration of higher than prophylactic doses, such as: (a) BMI ≥ 30 kg/m^2^ (OR: 1.7, 95% CI: 1–2.9, *p* = 0.0398), (b) body weight ≥ 73kg (OR: 1.9, 95% CI: 1.3–2.9, *p* = 0.0014), (c) metastatic disease (OR: 3.0, 95% CI: 2–4.5, *p* < 0.001), and (d) erythropoietin use (OR: 3.4, 95% CI: 2–6, *p* < 0.0001); for ORs the prophylactic dose administered was used as reference. Moreover, an increasing trend was observed for administration of higher than prophylactic doses, with regards to the clinical oncology setting (line of systemic anticancer treatment). In particular, the percentage of patients receiving higher than prophylactic doses was distributed as follows; preoperative: 23%, adjuvant: 34%, 1st line: 50%, 2nd line: 60%, and 3rd line: 65% (*p* = 0.0364).

### 2.7. Thrombotic and Bleeding Events

During the study, thrombotic events occurred in 16 of 426 patients (3.8%, 95% CI: 2.3–6.0%), 9 Deep Vein Thrombosis (DVT), 5 Pulmonary Embolisms (PE), and 2 Arterial Thrombosis Events (ATE). Twelve events were symptomatic and four incidental. The cancer types that had thrombotic events were: lung 5, breast 3, gastrointestinal 3, and other 5, while eleven of these patients had metastatic disease. With regards to the treatment related factors, 11 of 16 patients with thrombotic events received HTRCAs. Additionally, 20% of patients who received tamoxifen had thrombotic events. For patient-related factors, high BMI was a differentiating risk factor, as 25% of patients with thrombotic events had BMI ≥ 35 kg/m^2^. Within the group of patients without thrombotic events, only 5.6% had BMI ≥ 35 kg/m^2^. In terms of medical history, history of trauma was linked with thrombotic events (*p* = 0.0362). In terms of co-morbidities, two patients had cardiological issues, two had respiratory, and one had metabolic problems. The detailed results of the statistical analysis comparing patients with and without thrombotic events are presented in the Appendix (Table A3).

Out of 16 patients who experienced thrombotic events, 4 had a Khorana score ≥3 and received prophylactic doses. From the remaining 12 patients who had a Khorana score ≤2, 8 received prophylactic doses, and 4 higher doses, leading to OR: 0.3 (95% CI: 0.1–1.0, *p* < 0.05). Thus, patients who received higher than prophylactic doses had a 70% less odds to experience a thrombotic event.

**Figure 3:**
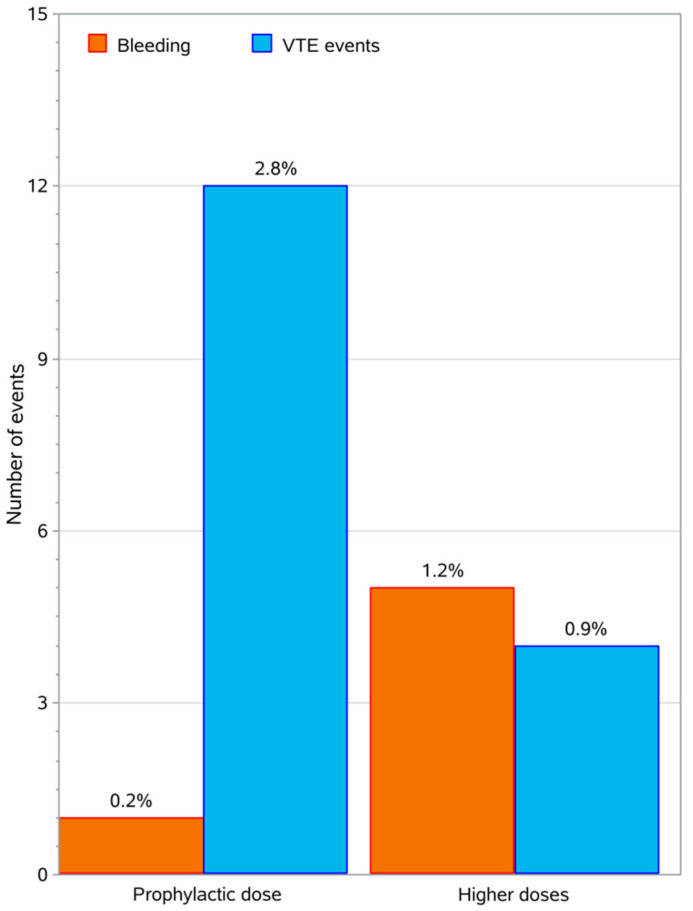
Bar chart with the VTEs and the bleeding events, grouped according to the anticoagulation dose. The percentage above the bars refers to the total number of patients.

Out of the 426 patients 6 (1.4%, 95% CI: 0.7–3.0%) presented with a bleeding event. All bleeding events were minor, 1 event was epistaxis, 1 hematuria, and 4 events were hemoptyses. Bleeding events occurred in 0.2% of patients on prophylactic doses and 1.2% of patients on higher doses. A graphical representation cumulatively showing the thrombotic events (VTEs) and bleeding events reported in this study in relation to the thromboprophylaxis dose administered is illustrated in Figure 3. The details of the statistical analysis for the bleeding events is presented in the Appendix (Table A4).

Moreover, with regards to the total study population (426 patients), twelve (4.0%) patients out of 304 with a Khorana score ≤2 had thrombotic events, while 4 (3.3%) patients with Khorana score ≥3 had such events (*p* > 0.05). Notably, thrombotic events has occurred in 2.8% of patients who received prophylactic doses (50% of the total participants) and 0.9% of patients who received higher doses. Thrombotic events occurred in 14 of 407 patients who received tinzaparin and in 2 of 10 patients who received other LMWHs.

## 3. Discussion

This observational, prospective, multicenter study enrolled 426 patients with active cancer, and aimed to record the thromboprophylaxis approach in clinical oncology practice. Thrombosis is an independent risk factor for mortality, especially during the first 4 chemotherapy cycles, and continuously increases during the ongoing treatment of cancer [24]. The risk of mortality from thrombosis is 47 times higher in cancer patients, compared to patients without cancer. A holistic approach to treat the patient and not only the disease is imposed since the times of Hippocrates; thus, prophylaxis from thrombotic events is a key parameter of this approach, being necessary not only to protect the patients’ survival and quality of life, but additionally to facilitate physicians to focus on active cancer therapy.

It was suggested that the Khorana score [19], despite being validated by many studies [25,26,27], does not take into consideration all risk factors associated with CAT. Cancer patients are fragile, usually of older age, have a poor performance status, with co-morbidities requiring polypharmacy, high incidence of renal impairment, and are exposed to treatment combinations with potentially nephrotoxic effects [28].

In this study, the efficacy and safety of thromboprophylaxis was recorded. We found evidence that thromboprophylaxis can reduce the number of VTE events, with no apparent increase in the incidence of major bleeding in patients with cancer. A patient characteristic analysis of patients with thrombosis and bleeding events was also performed. Factors found to be related to thrombosis events were: high BMI, history of trauma, and tamoxifen. Some characteristics linked to bleeding events were: age less than 65 years, history of thrombosis, high leukocytes count, and Khorana score ≥3.

The mechanisms underlying High Thrombotic Burden (HTB) development in cancer patients are complex and multifaceted. A complex coagulopathy develops in parallel with malignancy and is characterized by activation of clotting mechanisms, to different extent in different patients, and in different types of tumor. Tumor cells release procoagulant factors (i.e., Tissue Factor (TF), Cancer Procoagulant (CP), Factor VII), and microparticles (MP), which activate the coagulation cascade. Tumor cells also activate the host hemostatic cells (endothelial cells, leukocytes, and platelets), by either a release of soluble factors or by direct adhesion contact, thus, eliciting the expression of a procoagulant phenotype of these cells. In addition, the neutrophils can release neutrophil extracellular traps (NETs), and the adhesion of a large quantity of NETs to the vasculature might initiate thrombosis by providing a scaffold for platelet adhesion, activation, and thrombin generation [6].

Metastasis also contributes to thrombotic burden and a high percentage (up to 70%) of patients with metastatic disease depicted in our study. HTRCAs have a multifactorial contribution to the risk of thrombosis; induce vascular injury, lead to the release of prothrombotic particles and TF, increase thrombin potential, P-selectin and von Willebrand factor (vWF) activity, decrease protein C levels, and are also associated with decreased thrombus resolution. HTRCAs were used in patients with Khorana score ≥2 up to 61%, while in the total study population were used up to 67% [21].

Two ramdomized clinical trials (RCTs) performed analysis on prophylaxis with LMWHs for pancreatic cancer. The FRAGEM [29] trial reported a 3.4% risk for VTE (2/59 patients) and the CONKO-004 reported a 1.3% risk (2 from 160 patients) [30]. The risk for major bleeding was 3.4% (2/59) and 4.4% (7/160), respectively. None of the 59 patients with pancreatic cancer in our study experienced a thrombotic event and one patient had a minor bleeding event (risk 1.7%). The number of patients and events in all of these studies were low. In a meta-analysis of 6 studies (4315 patients) focusing on prophylaxis for lung cancer [31] the risk for VTE was reported as 4.0% and the pooled risk for major bleeding was 1.5%. The cohort of patients with lung cancer in our study had 107 patients, five had thrombotic events, and 4 experienced bleeding events, with a 4.7% and 3.7% risk, respectively. It was not possible to confirm statistically significant difference. From the RCTs studies targeting specific groups of patients that were at a high-risk for thrombosis, pooled results [20] of three RCTs studying thromboprophylaxis in cancer patients with Khorana score ≥3 (PHACS [32] SAVE-ONCO [33] and the PROTECHT [34] studies), reported a 3.3% risk for VTE in patients receiving LMWHs. The risk in the cohort of our study patients with Khorana score ≥3 was similar (3.3%, 4/122 patients). In summary, our study results are in line with the published literature.

In this study, the majority of the patients received thromboprophylaxis with LMWHs, which are also reported to possess potential anti-angiogenesis and anti-inflammatory effects [35]. The mechanism involves the release of TFPI, which subsequently inhibits the tissue factor (TF) that can be expressed on cancer cell surface [36]. TF activates both the coagulation cascade, a function that explains the link of cancer and thrombosis [37], and stimulates the production of growth factors and cytokines. The action of LMWHs further triggers the mechanism for additional inhibition of cancer cell-dependent platelet activation, and can inhibit selectin-dependent cell adhesion, as well as endothelial glycocalyx (stromal proteins). LMWHs enhance anti-inflammatory action by reducing tumor necrosis factor alpha (TNF-a) [38] and interferon, and indirectly inhibits PARs signaling (through the coagulation cascade) [35] and consequently, the vascular endothelial growth factor (VEGF) [39].

In the authors’ opinion, an individualized approach (using a large variety of risk factors) depicting the complexity of the situation in active cancer patients is needed for a clinical decision to apply either prophylactic or higher anticoagulation LMWHs dosage, irrespective of the Khorana score. Khorana score was found not to affect completely anticoagulant thromboprophylaxis dose decision. Remarkably, patients with a Khorana score ≥3 received prophylaxis for longer time periods. Additionally, the percentage of patients who received higher than prophylactic dose in the 1st, 2nd, and 3rd line treatment was almost double in comparison to patients in pre-operative and adjuvant treatment.

Recently, DOACs were investigated for thromboprophylaxis in cancer patients [20]; two studies reported results. In the AVERT trial in which apixaban was evaluated compared to placebo, 12 cases of VTE were reported (4.2% out of 288 patients) and in the modified intention-to-treat analysis, major bleeding occurred in 10 patients (3.5%). During the treatment period, major bleeding occurred in 6 patients (2.1%). The CASSINI trial evaluated the role of rivaroxaban in comparison to placebo [40]. The primary efficacy endpoint (diagnosis of VTE) occurred in 25 of 420 patients (5.95%), while major bleeding occurred in 8 of 405 (1.98%), and clinically relevant non-major bleeding occurred in 2.72% of them.

Both AVERT and CASSINI excluded certain groups of cancer patients [41], such as patients with low creatinine clearance (CrCl < 30mL/min), significant co-morbidities, bleeding diathesis, low platelets count, patients with Khorana score <2, and patients receiving chemotherapy treatment interacting with DOACs. Our study was a pragmatic one and enrolled all patients with active cancer (i.e., on chemotherapy treatment, with advanced or metastatic disease). In our study, 67 (16%) of the 426 patients enrolled had stomach or colorectal cancer and 16 patients had severe renal insufficiency. It is well-known that systemic anti-neoplasmatic therapies might interfere with DOACs, because inhibitors or inducers of P-glycoprotein and cytochrome p450 CYP3A4, influence the metabolism of DOACs and thus their efficacy and safety profiles [42]. Interestingly, in our study, 343 patients (82%) received chemotherapy with known drug–drug interactions (DDIs) with DOACs. As our study represents the clinical practice (real world data), no direct comparison is possible. DOACs could be an alternative approach, provided that there are no significant risk factors for bleeding and DDIs [20].

Guidelines [20] recommend that thromboprophylaxis for high-risk outpatients with active cancer (Khorana score ≥ 2 prior to starting a new systemic chemotherapy regimen), could be offered, provided that there are no significant risk factors for bleeding and no drug–drug interactions. In this multicenter study, it was revealed that the decision to administer CAT prophylaxis was not based only on the Khorana score, since additional factors were taken into consideration in patients with Khorana score ≤2 (Figure 2 and Table 4). All factors relevant to such decisions are grouped in the well-known categories of patient, cancer, treatment, and biomarker-related; in reality the thromboprophylaxis decision proved to be more complex and involved a multitude of parameters. At the second level, after the decision to administer CAT protection, a second decision related to the anticoagulation dosage had to be made. In our study, this decision was influenced by factors related to the patient body weight and BMI, the presence of metastases, as well as the administration of erythropoietin and the clinical oncology setting of anticancer treatment.

There are some recent data related to quality of life and compliance to anticoagulation, with LMWHs during prolonged treatment [43]. Our study had no special focus to evaluate patients’ quality of life; however, patients tolerated the anticoagulation treatment well, since no adherence issues were reported during the anticoagulation period (5.0 ± 3.1 months).

Our study had the limitations and advantages of a pragmatic study [44]. For example, the trial was designed in a broad range routine clinical practice, without specific focus on patients’ characteristics, thus, unknown bias could be introduced. There was no selection of patients into intervention and control groups; additionally the analysis included all participants in an intention-to-treat concept, among other potential limitations. However, in the author’s opinion, this study captured the real-life conditions in a routine clinical oncology setting. One of the strengths of our approach was the initial follow-up (12 months), while the study was continued for 12 additional months.

## 4. Materials and Methods

This was an observational, multicenter, prospective, study (ClinicalTrials.gov code: NCT03292107) that aimed to collect data regarding the thromboprophylaxis of CAT during daily clinical practice. Eighteen oncology departments throughout Greece participated in the study. The study was approved by the Bioethics and Scientific Committees of the participating hospitals. The inclusion criteria according to the study protocol were as follows: patients’ age ≥ 18 years, diagnosed with histologically confirmed solid tumor, under anticoagulation prophylaxis, and with signed informed consent form according to the Helsinki declaration. There was no specific guidance on thromboprophylaxis details from the study protocol; however, oncologists followed their individual/center practices based on the recommendations of ESMO and ASCO. Patients were followed-up initially for one year, in order to record their data regarding the efficacy and safety of anticoagulation thromboprophylaxis.

During their initial visit, demographics, somatometric, clinical parameters, and data related to medical history were collected. Subsequently, data related to chemotherapy regimens, anticoagulant use, and thromboprophylaxis safety and efficacy were recorded.

The primary aim of this study was to record the clinical practice of thromboprophylaxis. The secondary endpoints were the incidence of the VTE and bleeding events. The VTE events had to be objectively confirmed by compression ultrasonography or contrast venography (for deep venous thrombosis DVT) and computed tomography (CT scan, MRI scan, or pulmonary angiogram) or pulmonary ventilation/perfusion scan (for pulmonary embolism PE). Major bleeding was defined as any fatal bleeding or symptomatic bleeding in a critical area or organ; extra surgical site bleeding causing a fall in hemoglobin level 2 g/dL; or leading to transfusion of two or more units of whole blood or red cells, or surgical site bleeding that required a second intervention; or that was unexpected and prolonged or sufficiently large to cause hemodynamic instability [3]. Other clinically relevant non-major bleeding (CRNMB) defined as overt bleeding not meeting the criteria for major bleeding but associated with medical intervention, unscheduled contact (visit or telephone call) with a physician, (temporary) cessation of study treatment, or associated with discomfort for the patient, such as pain, or impairment of activities of daily life. All other bleeding events were classified as minor [45,46]. Follow-up period comprised from study enrollment up to 12 months. Main clinical data, the VTE risk score and pharmacological thromboprophylaxis use were provided by multiple sources of institutions’ medical records. VTE or bleeding events after discharge were obtained from outpatient visits’ medical records.

### Statistical Analysis

The statistical analysis was performed via the open source R language; Excel 2007 (Microsoft Corporation, Redmond, WA, USA) was used for data collection, conditioning, and preprocessing. Statistical tests for numerical parameters were performed via the Kruskal–Wallis test and comparisons of categorical parameters within groups were conducted via the chi-square test. The significance level for the study was set to 0.05. Since thrombotic and bleeding events are rather scarce and no sample size calculation was performed, we decided to register around 500 patients, in order to not exceed time and other resources, and simultaneously maintain similar population with other studies.

## 5. Conclusions

Cancer is a complex and omni-coagulable disease that requires dynamic and continuous assessment and management in terms of thromboprophylaxis, as thrombosis could be fatal and still remains the second leading cause of death in cancer patients. Oncologists are aware of the negative consequences of thrombosis in active cancer patients. Numerous factors affect the oncologists’ decision to protect their patients against CAT, besides the Khorana score. Administration of LMWHs in effective doses in active cancer patients receiving systemic chemotherapy is effective and safe.

## Figures and Tables

**Table 1:** Baseline characteristics of patients.

Patients Characteristics	Value
Number of patients	426
Male (%)	54.6%
Age (mean ± SD)	65.3 ± 11.2
Lung	107 (25.1%)
Pancreas	59 (13.9%)
Breast	37 (8.7%)
Stomach	36 (8.5%)
Ovarian	33 (7.8%)
Colorectal	31 (7.3%)
Bladder	24 (5.6%)
Prostate	14 (3.3%)
Testis	6 (1.4%)
Other*	79 (18.5%)

* Other non-listed solid tumors—head & neck, skin, brain, liver, unknown primary site, etc.

**Table 2:** Factors contributing to cancer-associated thrombosis (CAT) risk and anticoagulation approach.

Patients Characteristics	Value
BMI (mean ± SD)	26.1 ± 5.1
BMI ≥35 kg/m^2^	6.3%
Anemia (Hg < 10 g/L)	20.2%
PLT count ≥ 350 × 10^9^/L	37.1%
Leucocytes count > 11 × 10^9^/L	23.7%
Erythropoietin use	16.7%
Metastatic disease	69.5%
HTRCAs*	67.1%
Khorana score ≤ 1	43.9%
Khorana score = 2	27.5%
Khorana score ≥ 3	28.6%

HTRCAs*—High Thrombotic Risk Chemotherapy Agents.

**Table 3:** Distribution of patients according to cancer type and risk factors for CAT grouped as cancer, treatment, patient, and biomarker-related categories.

		Cancer	Treatment	Patient		Biomarker		
Primary Site	Patients (*N*, %)	Metastatic (*N*, %)	HTRCAs (*N*, %)	Age (≥65, *N*, %)	Comorbidities (*N*, %)	PLT Count (≥350 × 10^9^/L, *N*, %)	Anemia (Hg < 10 g/L, *N*, %)	Leucocytes (>11 × 10^9^/L, *N*, %)
**Lung**	107 (25.1%)	80 (74.8%)	70 (65.4%)	59 (55.1%)	27 (25.2%)	48 (44.9%)	26 (24.3%)	35 (32.7%)
**Pancreas**	59 (13.9%)	43 (72.9%)	55 (93.2%)	35 (59.3%)	16 (27.1%)	21 (35.6%)	11 (18.6%)	13 (22.0%)
**Breast**	37 (8.7%)	25 (67.6%)	13 (35.1%)	18 (48.7%)	5 (13.9%)	4 (10.8%)	4 (10.8%)	1 (2.7%)
**Stomach**	36 (8.5%)	29 (80.6%)	31 (86.1%)	24 (66.7%)	9 (25%)	14 (38.9%)	9 (25%)	6 (16.7%)
**Ovarian**	33 (7.8%)	19 (57.6%)	25 (75.8%)	19 (57.6%)	9 (27.3%)	12 (36.4%)	6 (18.2%)	5 (15.2%)
**Colorectal**	31 (7.3%)	23 (74.2%)	25 (80.7%)	18 (58.1%)	8 (25.8%)	7 (22.6%)	7 (22.6%)	4 (12.9%)
**Bladder**	24 (5.6%)	17 (70.8%)	19 (79.2%)	17 (70.8%)	8 (33.3%)	12 (50%)	5 (20.8%)	8 (33.3%)
**Prostate**	14 (3.3%)	12 (85.7%	1 (7.1%)	10 (71.4%)	3 (21.4%)	4 (28.6%)	4 (28.6%)	
**Testis**	6 (1.4%)	2 (33.3%)	6 (100%)	1 (16.7%)	1 (16.7%)	4 (66.7%)	0 (0%)	2 (33.3%)
**Other**	79 (18.5%)	47 (59.5%)	41 (51.9%)	38 (48.1%)	29 (36.7%)	32 (40.5%)	14 (17.7%)	27 (34.2%)

**Table 4:** Risk factors for CAT recorded during the study.

Risk Group	Factor
**Cancer Related**	Cancer type has a very high risk for CAT according to the Khorana score
	Cancer type has a high risk for CAT according to the Khorana score
	Cancer type is not included in the Khorana score
	Metastatic disease
**Treatment Related**	Use of HTRCAs during treatment
	Cancer surgery was performed
	Hospitalization of patient
	Use of erythropoietin
	Use of hormonotherapy (tamoxifen)
**Patient Related**	BMI ≥35 kg/m^2^
	Age >65 years
	Smoking
	Co-morbidities
	History of trauma or heart failure or thrombosis
**Biomarkers**	Pre-chemotherapy platelets count ≥350 × 10⁹/L
	Hemoglobin level <10 g/dL or use of erythropoietin
	Pre-chemotherapy leukocytes count >11 × 10⁹/L

## Appendix A

**Table A1:** Baseline characteristics for the patients with Khorana score ≤2.

Patients Characteristics	Value
Number of patients	304
Male (%)	50.7%
Age (mean ± SD)	65.5 ± 11.3
Lung	72 (23.7%)
Breast	37 (12.2%)
Colorectal	30 (9.9%)
Ovarian	25 (8.2%)
Pancreas	24 (7.9%)
Stomach	14 (4.6%)
Prostate	14 (4.6%)
Bladder	12 (4.0%)
Testis	4 (1.3%)
Other*	72 (23.7%)

* Other non-listed solid tumors: head and neck, skin, brain, liver, unknown primary site, etc.

**Table A2:** Risk factors for CAT and prophylactic approach in the cohort of patients with Khorana score ≤2.

Patients Characteristics	Value
BMI (mean ± SD)	26.1 ± 4.8
BMI ≥ 35 kg/m^2^	3.6%
Anemia (Hg <10 g/L)	12.5%
PLT count ≥ 350 × 10^9^/L	20.6%
Leucocytes count > 11 × 10^9^/L	11.2%
Erythropoietin use	15.6%
Metastatic disease	68.8%
HTRCAs	60.9%
Khorana score ≤ 1	47.0%
Khorana score = 2	53.0%

**Table A3:** Statistical comparisons between patients without thrombotic events vs. patients with thrombotic events. OR: Odds Ratio, LCL: 95% Lower Confidence Limit, UCL: 95% Upper Confidence Limit. Higher OR indicates less probability for thrombosis.

Parameter	*p*-Value	OR	LCL	UCL
Gender (Female vs. Male)	0.5170	1.4	0.5	3.9
Age (<65 years vs higher)	0.2162	0.5	0.2	1.5
BMI (<35 kg/m^2^ vs. higher)	**0.0018**	**5.6**	**1.7**	**18.8**
Smoking (No vs. Yes)	0.2014	2.1	0.7	6.6
History of thrombosis (No vs. Yes)	0.1919	3.8	0.4	33.0
History of trauma (No vs. Yes)	**0.0362**	**4.7**	**0.96**	**23.0**
History of surgery (No vs. Yes)	0.3796	1.6	0.6	4.3
History of comorbidities (No vs. Yes)	0.7005	1.2	0.4	3.6
Hospitalized Patient (No vs. Yes)	0.0525	3.1	0.9	10.2
Metastasis (No vs. Yes)	0.7410	0.8	0.3	2.5
Radiotherapy (No vs. Yes)	0.3795	1.6	0.6	4.5
HTRCAs (No vs. Yes)	0.8886	1.1	0.4	3.1
Agents for Erythropoiesis (No vs. Yes)	0.0682	Not applicable, one group had zero observations		
Leukocyte Count (pre chemotherapy) (Low vs. High)	0.4697	1.5	0.5	4.4
Hemoglobin (High vs. Low)	**0.0403**	All thrombotic cases had high hemoglobin		
PLT Count High (pre chemotherapy)	0.6221	0.8	0.3	2.2
Khorana Score (≤2 vs. ≥3)	0.7428	0.8	0.3	2.6
Agent for anticoagulation (prophylactic vs. higher dose)	**0.0413**	**0.3**	**0.1**	**1.0**

* Bold values indicate statistically significant differences *p* < 0.05.

**Table A4:** Statistical comparisons between patients with bleeding events vs. patients without bleeding events during the study duration for the categorical parameters.OR: Odds Ratio, LCL: 95% Lower Confidence Limit, and UCL: 95% Upper Confidence Limit. Higher OR indicates higher probability for bleeding.

Parameter	*p*-Value	OR	LCL	UCL
Gender (Femalevs. Male)	0.1544	0.2	0.1	2.0
Age (<65 years vs. higher)	**0.0353**	**7.3**	**0.8**	**62.9**
BMI (<35 kg/m^2^ vs. higher)	0.2957	0.3	0.04	2.99
Smoking (Novs. Yes)	0.2332	0.3	0.03	1.39
History of thrombosis (No vs. Yes)	**0.0075**	**0.1**	**0.01**	**0.9**
History of trauma (No vs. Yes)	0.6481	Not applicable, one group had zero observations		
History of surgery (No vs. Yes)	0.5461	1.7	0.3	9.3
History of comorbidities (No vs. Yes)	0.2027	0.4	0.1	1.8
History of renal Insufficiency (No vs. Yes)	0.1271	0.2	0.02	1.9
History of bleeding (No vs. Yes)	0.8655	Not applicable, one group had zero observations		
Hospitalized Patient (No vs. Yes)	0.3804	Not applicable, one group had zero observations		
Metastasis (No vs. Yes)	0.5452	0.5	0.1	4.5
Radiotherapy (No vs. Yes)	0.5383	1.9	0.2	16.8
HTRCAs (No vs. Yes)	0.3681	2.1	0.4	10.4
Use of Agents for Erythropoiesis (No vs. Yes)	0.2699	Not applicable, one group had zero observations		
Leukocyte Count (pre chemotherapy) (Low vs. High)	**0.0127**	**0.2**	**0.03**	**0.8**
Hemoglobin (High vs. Low)	0.2147	Not Applicable		
PLT Count High (pre chemotherapy)	0.1309	0.3	0.05	1.6
Khorana Score (≤2 vs. ≥3)	**0.0028**	**0.1**	**0.01**	**0.7**
Agent for anticoagulation (prophylactic vs. higher dose)	**0.0248**	Not applicable, one group had zero observations		

* Bold values indicate statistically significant differences *p* < 0.05.
